# Phase I study of cisplatin and nanoparticle albumin‐bound‐paclitaxel combined with concurrent radiotherapy in locally advanced esophageal squamous cell carcinoma

**DOI:** 10.1002/cam4.6205

**Published:** 2023-06-19

**Authors:** Hui Jiang, Qiaoqiao Li, Baoqing Chen, Mian Xi, Kanjiebubi Makelike, Shiliang Liu, Yonghong Hu, Yujia Zhu

**Affiliations:** ^1^ Department of Radiation Oncology, State Key Laboratory of Oncology in South China Collaborative Innovation Center for Cancer Medicine Sun Yat‐Sen University Cancer Center Guangzhou People's Republic of China

**Keywords:** dose escalation, esophageal squamous cell carcinoma, nanoparticle albumin‐bound‐paclitaxel, phase I study, radiotherapy

## Abstract

**Background:**

This phase I study aimed to assess the safety, dose‐limiting toxicity (DLT), maximum tolerated dose (MTD) and preliminary effect of nanoparticle albumin‐bound (nab)‐paclitaxel in combination with concurrent chemoradiotherapy in patients with locally advanced esophageal squamous cell carcinoma (ESCC).

**Methods:**

Patients with locally advanced ESCC who were ineligible or refused surgery were enrolled. Nab‐paclitaxel (60 mg/m^2^, 75 mg/m^2^, and 90 mg/m^2^) and cisplatin (25 mg/m^2^) were administered intravenously weekly on days 1, 8, 15, 22, and 29 on the basis of the 3 + 3 dose escalation method. The total dose of radiation was 50–64 Gy. The primary endpoint was the safety of chemotherapy.

**Results:**

The study enrolled 12 patients across three dose levels. No treatment‐related deaths occurred. One patient in the 60 mg/m^2^ dose level occurred dose‐limiting Grade 3 febrile neutropenia. No DLT was found in the 90 mg/m^2^ dose level thus the MTD was not reached. The phase II study's recommended dose was 75 mg/m^2^ based on the available preclinical and clinical data including pharmacokinetics, pharmacodynamics, efficacy, and toxicity. The frequent hematologic toxicities were leukocytopenia (Grade 1–2 of 66.7% and Grade 3–4 of 33.3%), neutropenia (Grade 1–2 of 91.7% and Grade 3–4 of 8.3%). Nonhematologic toxicities were mild and manageable. Overall response rate (ORR) of all patients achieved 100%.

**Conclusions:**

Weekly schedule of cisplatin and nab‐paclitaxel in combination with concurrent radiotherapy showed manageable toxicities and promising antitumor activity in patients with locally advanced ESCC. The recommended dose of nab‐paclitaxel for further studies is 75 mg/m^2^.

## 1 INTRODUCTION

As a highly aggressive malignancy, esophagus carcinoma becomes the seventh most prevalent malignancy and the sixth major cause of cancer‐related deaths worldwide.^1^ Histological types are mainly composed of adenocarcinoma and squamous cell carcinoma.^2^ However, esophageal squamous cell carcinoma (ESCC) occupies a dominant position all over the world, accounting for 90% in Eastern Europe and Asia.^3^ Owing to insufficient symptoms in early stage and the highly invasive innate character, esophageal cancer was frequently diagnosed with advanced or metastatic cases and relapse cases after operation. Nowadays, for those that cannot be excisable, locally advanced ESCC, concurrent chemoradiotherapy (CCRT) has been a recommended therapy.^4^, ^5^ The current 3‐year overall survival is approximately 30%–60%,^4^, ^6^, ^7^ which remains unsatisfactory. Oncologists have been seeking ways to improve the efficacy with tolerated toxicities. Among them, optimizing the chemotherapy regimen is one of the promising directions.

Although fluorouracil plus cisplatin (PF) is often used in locally advanced ESCC, it still has limited efficacy. Attempts have been made to take the advantage of next‐generation cytotoxic agents to improve survival. Paclitaxel is a class of cytotoxic chemotherapy drugs, which has always been an effective agent in the therapy of squamous cell carcinoma in multiple sites. In a prospective study of preoperative chemoradiotherapy for esophageal cancer, it was found that the pathological complete response (pCR) rate of ESCC patients with radiotherapy combined with paclitaxel plus carboplatin regimen was 49%, which was higher than the previous pCR rate of 30%–35% for preoperative CCRT with PF regimen.^8^ A retrospective paired analysis of ESCC showed that cisplatin plus docetaxel (DP) combined with radiotherapy improved survival compared with the PF regimen.^9^ Nevertheless, a prospective study of phase II showed that DP regimen combined with concurrent radiotherapy did not show the superiority in the overall survival (OS) in ESCC patients in comparison with the PF regimen. The 2‐year OS was 69% vs. 86% (*p* = 0.36), which may be related to a higher incidence of distant metastasis (33.3% vs. 9.8%, *p* = 0.008) in DP group and a higher rate of ≥ Grade 2 treatment toxicity (64% vs. 24%, *p* = 0.028) in DP group may have contributed to the fact that only 71% of the participants fulfilled the whole course of chemotherapy.^10^

The solvents used for conventional paclitaxel, for instance, polyoxyethylated castor oil and ethanol, can cause undesirable toxicities such as high‐sensitivity reaction and peripheral neuropathy, which require anti‐allergic drugs before administration, and can reduce the drug quantity and bioavailability.^11^ In summary, paclitaxel combined with radiotherapy for locally advanced ESCC is effective. If treatment toxicity can be better controlled and treatment compliance can be increased, the OS is expected to be improved.

As a solventless, human albumin‐stabilized formula of paclitaxel,^12^ nanoparticle albumin‐bound (nab)‐paclitaxel achieved faster and deeper penetration and slower elimination in the tissue.^13^ Preclinical study suggested that nab‐paclitaxel may get to tumor cells more efficiently with elevated accumulation than conventional solvent‐paclitaxel.^14^, ^15^ Based on the preclinical evidence, a great deal of trials have showed that nab‐paclitaxel can achieve a better tumor cell retention, less toxicity, and more promising antitumor response on non‐small cell lung carcinoma (NSCLC),^16^ cancer of pancreatic,^17^ cancer of breast,^18^ melanoma,^19^ and cancer of ovarian,^20^ comparing to the solvent‐paclitaxel. A prospective study found that carboplatin plus nab‐paclitaxel regimen was efficacious in patients with advanced NSCLC and achieved a notably improved overall response rate (ORR) versus conventional paclitaxel (33% vs. 25%, *p* = 0.005), especially in the type of squamous cell cancer (41% vs. 24%, *p* < 0.001). What's more, participants from the nab‐paclitaxel group occurred much weaker grade 3 toxicities, such as myalgia and neuropathy.^16^ A retrospective paired study, comparing the differences of ORR between the two kind of paclitaxel combined with cisplatin in ESCC, showed that the ORR rate of nab‐paclitaxel arm was higher (50% vs. 30%, *p* = 0.08).^21^

Based on the current data, it shows that nab‐paclitaxel seems effective in ESCC with tolerable toxicity. Nevertheless, we know little about the safety and preliminary efficacy of nab‐paclitaxel PF in combination with concurrent radiotherapy in locally advanced ESCC. The objectives of our study are to explore the safety, dose‐limiting toxicity (DLT), maximum tolerated dose (MTD) and preliminary efficacy of nab‐paclitaxel combined with CCRT in locally advanced ESCC.

## 2 MATERIALS AND METHODS

### 2.1 Patients

Our study is a phase I study of nab‐paclitaxel. From February 2019 to November 2019, 12 participants with locally advanced ESCC were enrolled in line with the eligible criteria: (1) histologically proven ESCC patients; (2) stage I to IVA disease (including metastatic cervical node), in accordance with the 8th edition of the American Joint Committee on Cancer (AJCC) staging system for esophageal carcinoma; (3) age of 18–70; (4) Eastern Cooperative Oncology Group performance status score ≤2; (5) normal functions of bone marrow, renal and liver.

Key exclusion criteria included: (1) distant metastasis (clinically confirmed or suspected); (2) any treatment before enrollment (other than exploratory surgery); (3) severe cardiovascular disease; (4) known allergy to study drugs; (5) other malignancies other than non‐melanin skin cancer within 5 years.

Sun Yat‐sen University Cancer Center (SYSUCC) ethics committee approved the protocol (Number B2018‐127‐01). Written informed consent was obtained from each patient. We carried out the study based on the requirements of Declaration of Helsinki. This study was registered on Chinese Clinical Trial Registry (ChiCTR1900021079).

### 2.2 Study design

**FIGURE 1:**
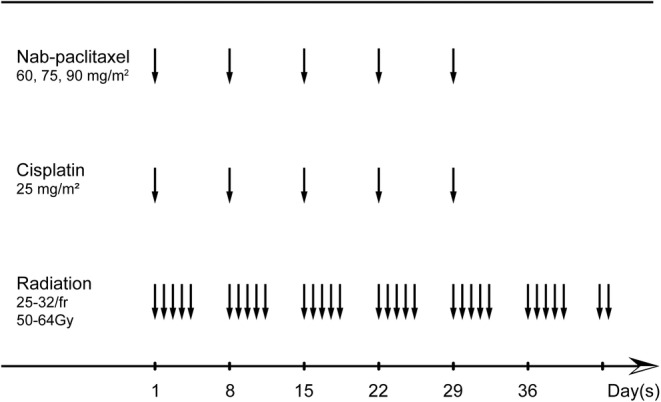
Treatment schedule. fr, fraction; Nab‐paclitaxel, nanoparticle albumin‐bound‐paclitaxel.

Treatment plan is illustrated in Figure 1.

#### 2.2.1 Chemotherapy

Chemotherapy included weekly administrations of cisplatin and nab‐paclitaxel on days 1, 8, 15, 22, and 29. 20 mL of 0.9% saline was used to dilute nab‐paclitaxel and then infused 30 min without pre‐medication of corticosteroid or antihistamine. The start dose of nab‐paclitaxel was set at 60 mg/m^2^. It was elevated by 15 mg/m^2^ per group. The weekly cisplatin dose was 25 mg/m^2^. 500 mL of 0.9% saline was used to dilute cisplatin and infused 2–6 h. To prevent renal toxicity, hydration is necessary.

#### 2.2.2 Radiotherapy

All the patients underwent intensity‐modulated radiation therapy (IMRT), which were calculated with the Monacle planning system. Vacuum bags were utilized to avoid differences in the daily radiotherapy. Radiotherapy treatment planning was conducted on the basis of recent CT outcomes. Gross tumor volume (GTV) included the primary esophagus disease and metastatic regional lymph node discovered by image inspection. GTV and 0.5–0.8 cm margins were delineated as planning target volume (PTV). The total radiation dose was 50–64 Gy in 25–32 fractions over 5–7 weeks. The dose constraints were as followed: the maximum spinal cord ≤50 Gy, the mean lung dose ≤20 Gy, the mean liver dose ≤25 Gy, the maximum gastrointestinal ≤56 Gy.

### 2.3 DLT and dose‐escalation

The occurrence of the following toxicities within first 6 weeks of chemoradiotherapy was considered a DLT: (1) absolute neutrophil count <1.0 × 10^9^/L lasting for at least 4 days; (2) absolute neutrophil count <1.0 × 10^9^/L accompanied by fever; (3) platelet count <50 × 10^9^/L; (4) Grade 3 sensory neuropathy; (5) Grade ≥3 non‐hematological toxicities, such as diarrhea, hand‐foot syndrome, oral mucositis, vomiting, anorexia, electrolyte disorders, nausea, liver function impairment, etc.; (6) treatment delayed for more than 1 week due to drug‐related side effects. Three participants were included into the starting dose level (60 mg/m^2^). If none of the three participants occurred DLT, another three patients received treatment at next level. Given that one of the three participants occurred DLT, another three additional participants would be enrolled. If one or two out of the six participants occurred DLT, the dose would be escalated to the next level. If two out of three or at least three out of the six participants occurred DLT, further dose escalations would be terminated, and the previous dose level would be defined as the MTD.

### 2.4 Efficacy and toxicity evaluation

The clinical response of each participant was evaluated by endoscopy and CT. Evaluation of treatment response was performed in line with the RECIST version 1.1. Complete response (CR) signified complete tumor disappearance and absence of viable cells in biopsy tissue samples. Partial response (PR) signified a reduction in the sum of target disease of ≥30%. Progressive disease (PD) signified a ≥ 20% increase in the primary tumor. Stable disease (SD) was between PR and PD. In addition, toxicity was evaluated according to the Common Terminology Criteria for Adverse Events (CTCAE) version 5.0.

### 2.5 Endpoints and statistical analysis

The mainly purpose was to assess the safety of nab‐paclitaxel in combination with radiotherapy and to confirm the phase II recommended dose. The secondary endpoints were ORR, disease control rate (DCR), progression‐free survival (PFS), and OS. Patient characteristics, treatments, and safety were evaluated by descriptive statistic method. Mean value and standard deviation were used for continuous data, while frequency and percentage distribution for discrete data. PFS was defined as time from the first dose of study treatment to the first documented disease progression per RECIST version 1.1. OS was defined as time from the first dose of study treatment to death. OS and PFS was estimated using Kaplan–Meier model. We analyzed data with Statistical Package for Social Sciences version 19.0 software.

## 3 RESULTS

### 3.1 Patient characteristics

Twelve patients were included from February 2019 to November 2019. All patients were available to assess toxicity and response. Baseline characteristics are shown in Table 1. It included 11 males and one female, ranging from 46 to 69 years (median, 57.5 years). Most of the participants (66.7%) were smokers. All of the participants were diagnosed with locally advanced ESCC, with the primary esophagus tumor mainly located in the upper thoracic region (58.3%). A total of 11 patients (91.7%) completed the treatment as planned. One patient in the 60 mg/m^2^ terminated the chemotherapy due to myelosuppression. Full dose of radiotherapy was carried out in all of the participants, and the average dose was 60 Gy.

**TABLE 1:** Baseline characteristics of patients.

Characteristics	Dose‐escalation			Total (*N* = 12)
	60 mg/m^2^ (*N* = 6)	75 mg/m^2^ (*N* = 3)	90 mg/m^2^ (*N* = 3)	
Age, years				
Median	61	50	56	57.5
Range	46–67	50–60	52–69	46–69
Sex				
Male	5	3	3	11
Female	1	0	0	1
ECOG Performance status				
0	0	0	0	0
1	6	3	3	12
Smoking status				
Yes	5	1	2	8
No	1	2	1	4
Drinking status				
Yes	5	0	1	6
No	1	3	2	6
AJCC clinical stage				
II	1	1	0	2
III	3	2	2	7
IVA	2	0	1	3
Location of primary tumor				
Cervical Upper thoracic Middle thoracic Lower thoracic Multi‐sites	0 4 1 1 0	1 0 1 0 1	0 3 0 0 0	1 7 2 1 1
Radiotherapy dose (Gy)				
Median	60	60	60	60
Range	55.9–60	60–63.8	60–60	55.9–63.8

Abbreviations: ECOG, Eastern Cooperative Oncology Group; AJCC, American Joint Committee on Cancer.

### 3.2 Efficacy and survival

**FIGURE 2:**
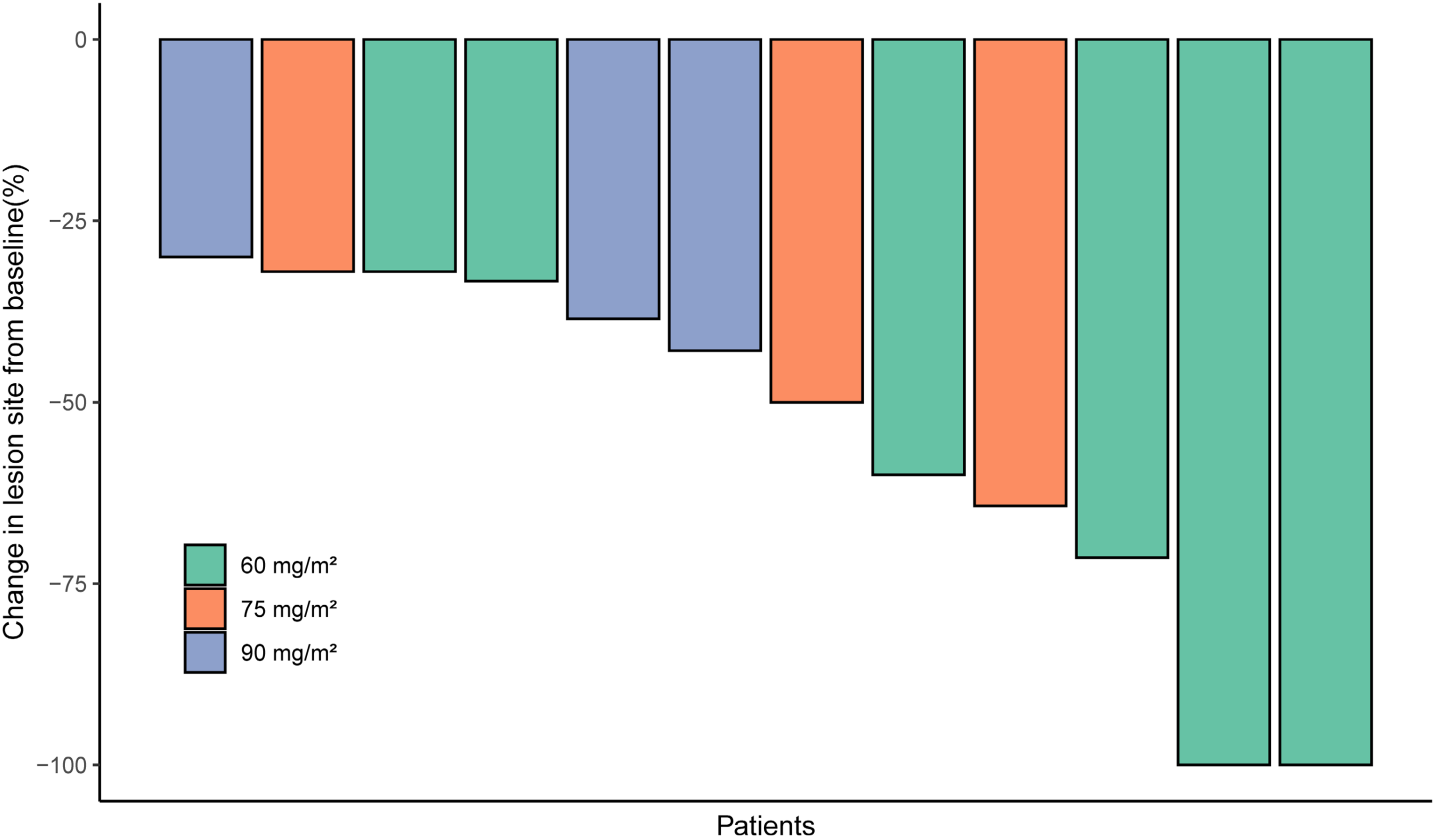
Waterfall plot of the maximal percentage reduction from baseline of the target lesions.

**FIGURE 3:**
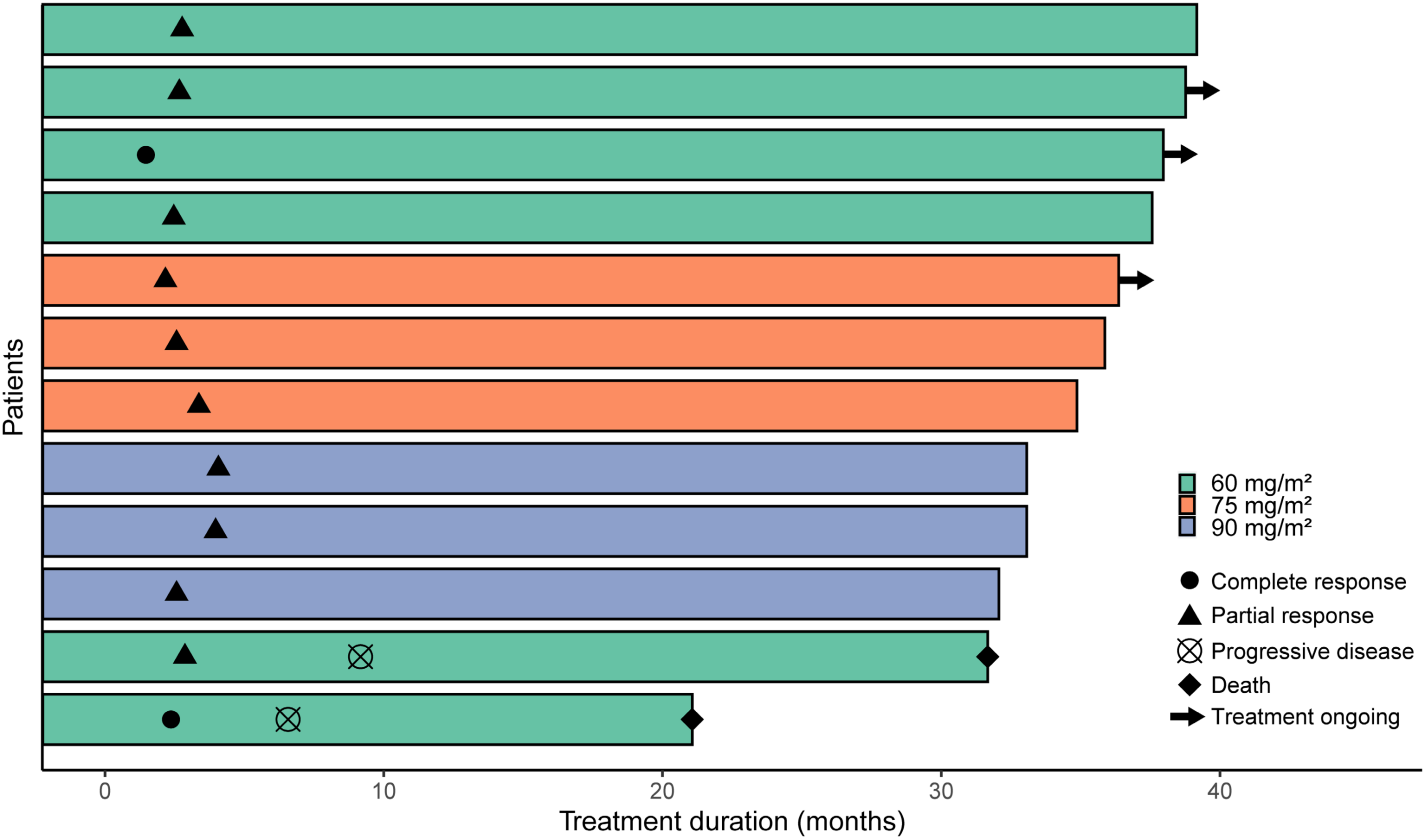
Duration of treatment. Each individual patient treatment time is plotted from time of first dose based on the dose level.

**FIGURE 4:**
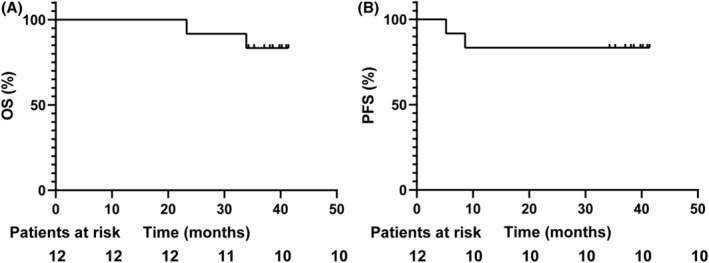
(A) Overall survival and (B) Progression‐free survival (Kaplan–Meier curve) of all patients. PFS, progression‐free survival; OS, overall survival.

All of the 12 participants were enrolled for efficacy analysis. Two (16.7%) participants from the 60 mg/m^2^ dose level experienced CR. The other 10 (83.3%) patients experienced PR. The ORR and DCR of all patients were both 100%. Detailed treatment response is listed in Table 2. A descriptive waterfall plot of the tumor regression is shown in Figure 2. Duration of exposure and RECIST evaluation were listed in Figure 3. After the median follow‐up of 38.1 months (range from 36.9–39.3), the 1‐year OS is 100%, the 2‐year OS is 91.7% (91.6–91.8%), the 3‐year OS is 83.3% (83.2–83.4%). The 1‐year, 2‐year and 3‐year PFS are both 83.3% (83.2–83.4%) (Figure 4). As of the last follow‐up time, two patients died due to the progress of the disease, while 10 patients still survived. Among them, two patients with primary treatment response following CRT for PR received oral medication (S‐1 or Apatinib) shortly after the initial examination until the final follow‐up, with the duration of treatment being 35 and 14 months, respectively. One CR patient has continued to take S‐1 orally to date due to personal preference. One PR patient received cisplatin and paclitaxel chemotherapy for two courses because of residual lymph node. Three PR patients received a short course of oral chemotherapy (S‐1 or Capecitabine) for 1–2 months after the initial review, and the drug was stopped when the next review was stable. Three PR patients received no subsequent treatment and were only routinely monitored.

**TABLE 2:** Response and survival data.

	Dose‐escalation			Total (*N* = 12)
	60 mg/m^2^ (*N* = 6)	75 mg/m^2^ (*N* = 3)	90 mg/m^2^ (*N* = 3)	
Response				
Complete response	2	0	0	2
Partial response	4	3	3	10
Stable disease	0	0	0	0
Progressive disease	0	0	0	0
ORR, %	100	100	100	100
DCR, %	100	100	100	100
OS, %				
1‐year	–	–	–	100
2‐year	–	–	–	91.7 (91.6–91.8)
3‐year	–	–	–	83.3 (83.2–83.4)
PFS, %				
1‐year	–	–	–	83.3 (83.2–83.4)
2‐year	–	–	–	83.3 (83.2–83.4)
3‐year	–	–	–	83.3 (83.2–83.4)

Abbreviations: DCR, disease control rate; ORR, overall response rate; OS, overall survival; PFS, progression‐free survival.

### 3.3 Toxicity

All patients were assessable for toxicity. No patient died due to the treatment‐related side effect. In addition, one patient in the 60 mg/m^2^ dose level had dose‐limiting grade 3 febrile neutropenia. Because of this, three additional patients were included at that dose level. No DLT was reported in the other patients of the study. The frequent hematologic toxicities were leukocytopenia (Grade 1–2 of 66.7% and Grade 3–4 of 33.3%), neutropenia (Grade 1–2 of 91.7% and Grade 3–4 of 8.3%), thrombocytopenia (Grade 1–2 of 8.3% and no Grade 3–4) and anemia (Grade 1–2 of 41.7% and no Grade 3–4). Nonhematologic toxicities were both mild and manageable. The most common type was radiation‐induced esophagitis (Grade 1–2 of 91.7% and no Grade 3–4). Others included diarrhea (Grade 2 of 8.3%), vomiting (Grade 1 of 8.3%), cough (Grade 1 of 8.3%), liver function injury (Grade 1 of 41.7%), loss of weight (Grade 1 of 25%). Generally, the treatment‐related toxicity was moderate and manageable (Table 3). No DLT was found in the 90 mg/m^2^ dose level cohort thus the MTD was not reached. The phase II study's recommended dose was established at 75 mg/m^2^ based on the available preclinical and clinical data including pharmacokinetics, pharmacodynamics, efficacy during the dose‐escalation phase.^13^, ^16^, ^21^ In summary, participants tolerated nab‐paclitaxel PF combined with radiotherapy very well. Clinicians should be aware of transient fever in the treatment.

**TABLE 3:** Acute toxicity during chemoradiotherapy.

Toxicity	60 mg/m^2^ (*n* = 6)				75 mg/m^2^ (*n* = 3)				90 mg/m^2^ (*n* = 3)				Total (*n* = 12)			
	G 1	G 2	G 3	G 4	G 1	G 2	G 3	G 4	G 1	G 2	G 3	G 4	G 1	G 2	G 3	G 4
Hematologic																
Leukocytopenia	0	4	1	0	0	2	1	0	0	2	2	0	0	8	4	0
Neutropenia	2	2	1	0	3	0	0	0	2	2	0	0	7	4	1	0
Thrombocytopenia	0	0	0	0	1	0	0	0	0	0	0	0	1	0	0	0
Anemia	0	2	0	0	2	0	0	0	1	0	0	0	3	2	0	0
Nonhematologic																
Esophagitis	1	5	0	0	0	2	0	0	1	2	0	0	2	9	0	0
Esophageal fistula	0	0	0	0	0	0	0	0	0	0	0	0	0	0	0	0
Pneumonia	0	0	0	0	0	0	0	0	0	0	0	0	0	0	0	0
Diarrhea	0	0	0	0	0	1	0	0	0	0	0	0	0	1	0	0
Vomiting	0	0	0	0	1	0	0	0	0	0	0	0	1	0	0	0
Cough	1	0	0	0	0	0	0	0	0	0	0	0	1	0	0	0
Liver function injury	2	0	0	0	2	0	0	0	1	0	0	0	5	0	0	0
Kidney function injury	0	0	0	0	0	0	0	0	0	0	0	0	0	0	0	0
Loss of weight	1	0	0	0	1	0	0	0	1	0	0	0	3	0	0	0

Abbreviation: G, grade.

## 4 DISCUSSION

Paclitaxel antitumor drugs include paclitaxel, docetaxel, paclitaxel liposomes, and nab‐paclitaxel, which belongs to the new anti‐microtubule drugs. Notably, nab‐paclitaxel combines the drug paclitaxel with albumin, which requires no special solvent, is easy to use, has low toxicity and increases antitumor activity. Among them, nab‐paclitaxel was firstly approved in the United States for the therapy of breast carcinoma in 2005.^22^ It has been approved as a first‐line therapy for metastatic NSCLC since 2012.^16^ In 2013, in the field of metastatic pancreatic cancer, nab‐paclitaxel plus gemcitabine were used as the first‐line therapy.^23^

Nab‐paclitaxel exploits endogenous albumin transmission pathway to enhance the transportation across the monolayer endothelial cells and increase the penetration of paclitaxel to tumor cells, when compared to the conventional paclitaxel.^14^ It has been found that nab‐paclitaxel enters the swelling through interaction with the secreted protein acidic and rich in cysteine (SPARC). In the cells of tumor, the expression of SPARC protein is related to the clinical response rate of nab‐paclitaxel.^24^ Besides, Yelong Chen et al. demonstrated that SPARC protein expression and mRNA replication in esophagus tumor tissues were six times higher than the normal tissue in the surrounding, and this high expression was positively correlated with metastasis of ESCC,^25^ which gives us a lot of confidence that we can use nab‐paclitaxel as part of a comprehensive therapy model in the cure of locally advanced ESCC.

Clinical studies of nab‐paclitaxel in the advanced or recurrent ESCC have been conducted in several centers (Table 4).^21^, ^26^, ^27^, ^28^, ^29^, ^30^, ^31^, ^32^ Dai et al. carried out a study to explore the efficacy and toxicity of nab‐paclitaxel PF as a first‐line regimen for patients with metastatic ESCC. Patients received nab‐paclitaxel 250 mg/m^2^ and cisplatin 75 mg/m^2^ i.v. on day 1, every 3 weeks. They found that the ORR achieved 60.6% and the DCR achieved 87.9%. The median PFS was 6.2 months and the median OS was 15.5 months.^27^ Besides, Mao et al. found that weekly nab‐paclitaxel with three‐weekly cisplatin is safe and effective as a neoadjuvant chemotherapy regimen for patients with locally advanced ESCC.^29^ Nowadays, in a numerous of esophageal clinical trials, nab‐paclitaxel is being used as one of the combinations of chemotherapy combined with immunotherapy.^31^, ^32^ So far, this is the first study on the toxicities and preliminary treatment outcomes of weekly nab‐paclitaxel plus weekly cisplatin in combination with concurrent radiotherapy in locally advanced ESCC, especially in the immunotherapy era.

**TABLE 4:** Studies on nab‐paclitaxel with or without radiotherapy in esophagus carcinoma.

Author	Year	Type	N	Histology	Nab‐paclitaxel	Other treatments	Toxicity (≥ Grade 3)	Survival
Thomas^26^	2008	Prospective	1	‐	260 mg/m^2^ 300 mg/m^2^ (MTD) 340 mg/m^2^ D1, Q3W	Gemcitabine	–	SD
Dai^27^	2013	Prospective	33	Sq	250 mg/m^2^ D1, Q3W	Cisplatin	Neutropenia, 6.0% Vomiting, 3.0% Neuropathy, 3.0%	ORR: 60.6% DCR: 87.9%
Shen^28^	2015	Retrospective	29	Sq	100–150 mg/m^2^ D1, 8, Q3W	Capecitabine or Oxaliplatin	Leucopenia, 24.2% Nausea/vomiting, 3.4%	ORR: 37.0% DCR: 44.4%
Liu^21^	2016	Retrospective	32	Sq	125 mg/m^2^ D1, 8, Q3W	Cisplatin	Leukopenia, 37.5% Neutropenia, 37.5% Febrile Neutropenia, 18.8% Thrombocytopenia, 18.8% Anemia, 28.1% Anorexia, 12.5% Nausea/vomiting, 6.3% Diarrhea, 6.3%	ORR: 50.0% DCR: 81.0%
Mao^29^	2016	Prospective	35	Sq	100 mg/m^2^ D1, 8, 22, 29	Cisplatin Surgery	Anemia, 8.6% Neutropenia, 11.4% Thrombocytopenia, 5.7% Febrile neutropenia, 8.6% Nausea/vomiting, 20%	pCR: 13.3% 1‐year OS: 90.0% 2‐year OS: 70.0% 3‐year OS: 43.3%
Pang^30^	2018	Prospective	17	Sq	60 mg/m^2^ D1, 8, 15, 22 Q4W	Cisplatin Radiotherapy	Leukopenia, 11.8% Anemia, 5.9% Esophagitis, 17.6% Anorexia, 5.9% Nausea, 5.9% Dysphagia, 5.9% Pneumonitis, 11.8% Cough, 11.8%	ORR: 88.2% 3‐year OS: 17.0% 3‐year PFS: 35.0%
Cheng^31^	2022	Prospective	23	Sq	260 mg/m^2^ D1, Q3W	Camrelizumab Carboplatin Surgery	Neutropenia, 39.1% Leukopenia, 8.7%	pCR: 25.0% ORR: 90.5% DCR: 100%
Li^32^	2022	Prospective	60	Sq	100 mg/m^2^ D1, 8, 15, Q3W	Camrelizumab Carboplatin Surgery	Leukopenia, 50.0% Anemia, 6.7% Thrombocytopenia, 6.7%	pCR: 39.2%
Currentstudy	2022	Prospective	12	Sq	60 mg/m^2^ 75 mg/m^2^ 90 mg/m^2^ D1, 8, 15, 22, 29	Cisplatin Radiotherapy	Neutropenia, 8.3% Leukopenia, 33.3%	ORR: 100% DCR: 100% 1‐year OS: 100% 2‐year OS: 91.7% 3‐year OS: 83.3% 1‐year PFS: 83.3% 2‐year PFS: 83.3% 3‐year PFS: 83.3%

Abbreviations: D, day; DCR, disease control rate; MTD, maximum tolerated dose; Nab‐paclitaxel, nanoparticle albumin‐bound‐paclitaxel; ORR, overall response rate; OS, overall survival; pCR, pathologically complete response; PFS, progression‐free survival; SD, stable disease; Sq, squamous cell carcinoma; Q3W, every 3 weeks; Q4W, every 4 weeks.

In our present study, no new safety event was observed and the adverse event was comparable to those previous studies.^21^, ^27^ Hematologic toxicities were the major adverse events. All of them were manageable with adequate symptomatic treatment or dose interruption or reduction, which indicated that nab‐paclitaxel in combination with concurrent radiotherapy was tolerated in patients with locally advanced ESCC. Besides, though no anti‐allergic medications before using nab‐paclitaxel were given, no allergic events were observed, which demonstrated a satisfiable tolerability of this formulation. This was also consistent with the previous findings. In spite of not reaching the MTD in our research, we still recommend further investigation using nab‐paclitaxel at a dose of 75 mg/m^2^. This conclusion was reached on the basis of an amount of preclinical and clinical research conducted on nab‐paclitaxel. It was initially used for the treatment of breast cancer. A significant phase III study for metastatic breast carcinoma employed a dose regimen of 260 mg/m^2^ used as a single agent once every 21 days, which had been shown to be safe and effective.^22^ Afterwards, nab‐paclitaxel was gradually introduced into the treatment of esophageal cancer. Based on the results of a retrospective study,^21^ nab‐paclitaxel (125 mg/m^2^, Day 1, Day 8, every 21 days) combined with cisplatin was recommended as a first‐line therapy for advanced esophageal cancer. And now, in many prospective studies to explore preoperative neoadjuvant therapy for locally advanced esophageal cancer, chemotherapy regimen based on nab‐paclitaxel (260 mg/m^2^, Day 1, every 21 days) has been adopted.^31^, ^33^, ^34^, ^35^ Above all, the cumulative dose of nab‐paclitaxel in esophageal cancer over a three‐week period usually does not exceed 250–260 mg/m^2^. Besides, paclitaxel had been found to exhibit a unique mechanism of radio‐sensitization. It could induce apoptosis and promote re‐oxygenation of tumor cells through arresting the cell cycle at the G2/M phase. Radiotherapy mainly targets cells in the G1, M, and G2 phases and has little effect on cells in the S phase, whereas chemotherapy has a strong effect on cells in the G1/S phase.^36^, ^37^ The two treatments complement each other. According to these findings, combined with our study results, if 90 mg/m^2^ per week is used, then the cumulative dose in 3 weeks will reach 270 mg/m^2^. After analysis and discussion with pharmacological experts, we hold the opinion that the concurrent nab‐paclitaxel dose of 90 mg/m^2^ per week in combination with radiotherapy may increase some of the unpredictable toxicities, especially in the cases with radiotherapy. We recommend the use of weekly nab‐paclitaxel 75 mg/m^2^ PF and concurrent radiotherapy and look forward to exploring the efficacy and toxicity in further studies.

Up to now, there was few robust evidence of the antitumor effect of nab‐paclitaxel combined with radiotherapy in locally advanced ESCC. In a pilot trial, patients received radical radiotherapy and concurrent chemotherapy including weekly nab‐paclitaxel 60 mg/m^2^ and weekly cisplatin 25 mg/m^2^, administered on days 1, 8, 15, and 22 of each 28‐day cycle. After a median follow‐up of 20.47 months, it reported that ORR was 88.2%, the 3‐year PFS was 17% and the 3‐year OS was 35%.^30^ Taking as reference the response rate in this trial, the effectiveness of nab‐paclitaxel in our study was inspiring, with the ORR and DCR of 100%. After the median follow up of 38.1 months, the 1‐year OS is 100%, the 2‐year OS is 91.7%, the 3‐year OS is 83.3%. The 1‐year, 2‐year and 3‐year PFS are 83.3%. Meanwhile, it is noteworthy that two patients have CR at the dose level of 60 mg/m^2^, which is significant in that a lower dose level might be employed in the maintenance treatment until disease progression, or intolerable toxicities henceforth.

In a stage of immunotherapy, many prospective phase III studies with immune checkpoint inhibitors have demonstrated that anti‐program death‐1 antibodies can be recommended as a standard second‐line therapy for advanced ESCC.^38^, ^39^ As a result, the comprehensive management of this high‐aggressive malignant tumor is changed. Nevertheless, there was limited benefit in response and survival for an unselected patient, and chemotherapy was still of significant value. Since nab‐paclitaxel becomes one of the efficacious cytotoxic drugs against ESCC, the antitumor response achieved in our study and the improved tolerance support future application of nab‐paclitaxel combined with radiotherapy and other antitumor agents in locally advanced ESCC patients.

Our study provides robust evidence that weekly nab‐paclitaxel‐based chemoradiotherapy is safe and effective for patients with locally advanced ESCC. Due to the nab‐paclitaxel's low toxicity, more patients might complete the full course of chemoradiotherapy. However, we also have to admit that there exist few limitations. Firstly, the sample size of this study was relatively small; hence, future study in larger samples is urgently needed. Secondly, due to geographical factor, only patient with squamous cell carcinoma were enrolled in our study. Therefore, the conclusion is only applicable to ESCC.

## 5 CONCLUSIONS

Weekly schedule of cisplatin and nab‐paclitaxel combined with concurrent radiotherapy showed manageable toxicities and promising antitumor effect in patients with locally advanced ESCC. The recommended dose of nab‐paclitaxel for further studies is 75 mg/m^2^. A phase II study is currently undergoing according to the results observed in this study.

## AUTHOR CONTRIBUTIONS

**hui jiang:** Data curation (equal); investigation (lead); methodology (equal); resources (equal); software (equal); writing – original draft (lead). **Qiao‐Qiao Li:** Data curation (equal); formal analysis (equal); methodology (equal); resources (equal); validation (equal). **Baoqing Chen:** Data curation (equal); investigation (equal); methodology (equal); resources (equal); writing – review and editing (equal). **Mian Xi:** Data curation (equal); investigation (equal); validation (equal). **kanjiebubi makelike:** Investigation (equal); resources (equal). **shiliang liu:** Investigation (equal); resources (equal). **yonghong hu:** Investigation (equal); resources (lead). **Yujia Zhu:** Conceptualization (lead); data curation (equal); formal analysis (equal); funding acquisition (equal); methodology (equal); project administration (equal); resources (equal); supervision (equal); validation (equal); writing – original draft (equal); writing – review and editing (equal).

## FUNDING INFORMATION

The Cspc Ouyi Pharmaceutical Co. Ltd.

## CONFLICT OF INTEREST STATEMENT

None.

## ETHICS STATEMENT

The protocol was reviewed and approved by the Sun Yat‐sen University Cancer Center (SYSUCC) ethics committee (Number B2018‐127‐01).

## PATIENT CONSENT

Each participant signed an informed consent.

## CLINICAL TRIAL REGISTRATION

Chinese Clinical Trial Registry (ChiCTR1900021079)

## Data Availability

The data utilized in the study can be obtained from the corresponding author on a reasonable request.
